# Designing CITOBOT: A portable device for cervical cancer screening using human-centered design, smart prototyping, and artificial intelligence

**DOI:** 10.1016/j.csbj.2024.11.018

**Published:** 2024-11-14

**Authors:** Marcela Arrivillaga, Paula C. Bermúdez, Juan Pablo García-Cifuentes, Hernán Darío Vargas-Cardona, Daniela Neira, Maria del Mar Torres, Mérida Rodríguez-López, Daniela Morales, Bleider Arizala

**Affiliations:** a Pontificia Universidad Javeriana - Cali, Calle 18, 118–250 Cali, Valle, Colombia; b Red de Salud Ladera ESE - Cali, 5C 39–51, Cali, Valle, Colombia; c Universidad Icesi, Cali, Calle 18, 122–135 Cali, Valle, Colombia

**Keywords:** Cervical cancer screening, Human-centered design, Artificial intelligence, Portable device, Cervical imaging, Medical device innovation

## Abstract

Cervical cancer remains a leading cause of mortality in its invasive stages, presenting a significant global public health challenge, particularly in low- and middle-income countries. Despite technological advancements that have improved the quality of cervical images captured during visual inspections, several challenges persist. This article presents key findings from the CITOBOT-COL translational research project, a large-scale initiative focused on designing CITOBOT as a portable cervical cancer screening device. We detail the comprehensive technological development of CITOBOT, guided by a human-centered design approach, smart prototyping, and the integration of AI. Over four design iterations, we developed and refined CITOBOT v4, a portable device. Prototypes were validated through focus groups and testing by experts in cervical cancer prevention, gynecology, nursing, software, artificial intelligence, computer engineering, and public health, utilizing various anatomical models at the Simulated Hospital Laboratory of Pontificia Universidad Javeriana Cali, Colombia. Additionally, we developed AI algorithms using the Inception V3 network, optimized with Transfer Learning and Fine Tuning, for cervical image classification and offline-operating software that guides the physician through the examination and provides a risk assessment for cervical cancer. Feedback was crucial in assessing and refining the device's functionality, focusing on capturing high-quality cervical images. The development of CITOBOT v4 highlights the importance of fostering innovation in resource-limited settings, offering an effective solution to improve cervical cancer screening and potentially save lives in vulnerable communities.

## Graphical abstract

**Figure:**
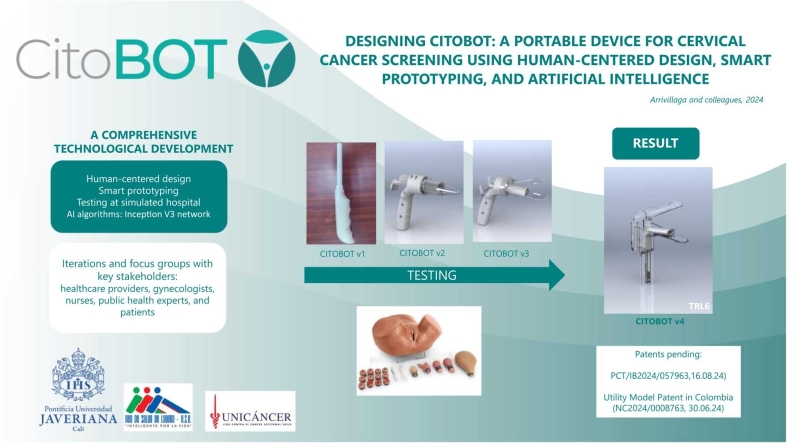

## 1 Introduction

Cervical cancer remains one of the leading causes of mortality in its invasive stage, representing a significant global public health challenge, particularly in low- and middle-income countries [1]. This cancer is largely preventable, and early detection is crucial in improving survival rates and quality of life [2]. Globally, cervical cancer ranks as the fourth most common cancer among women, with an estimated 662.301 new cases and 348,874 deaths reported in 2022. Notably, approximately 90 % of these cases and deaths occurred in low- and middle-income regions [3]. In Colombia, during the same period, an estimated 4572 new cases were reported, with an age-standardized incidence rate of 13.7 per 100,000 women and 2435 deaths, positioning cervical cancer as the third most common malignancy in terms of incidence and mortality among women [4].

The primary etiological agent of cervical cancer is the human papillomavirus (HPV) [5]. Currently, vaccines are available that provide effective prevention, mainly two prophylactic vaccines with high efficacy against HPV types 16 and 18, which are responsible for approximately 70 % of cervical cancer cases worldwide. Additionally, a nonvalent vaccine targets five more oncogenic HPV types, offering coverage for up to 90 % of all cases when combined with HPV-16 and 18 [6].

Several cervical cancer screening methods are available, including the Papanicolaou test (cytology), visual inspection with acetic acid (VIA), and HPV DNA testing. The Papanicolaou test remains the most used screening method. However, its effectiveness is contingent on the availability of laboratory infrastructure and skilled personnel for sample processing and analysis resources often limited in low-resource settings [7]. VIA, which involves the application of acetic acid (3 % to 5 %) to identify acetowhite areas that may indicate precancerous lesions, is more accessible but requires trained personnel for interpretation, introducing subjectivity and a higher risk of false positives [8].

Increasingly, HPV DNA testing is the global standard for cervical cancer screening due to its superior accuracy. With a reported sensitivity of 94 % and a specificity of 88 %, HPV DNA testing outperforms both cytology (sensitivity: 62.5–72.9 %, specificity: 90.3–96.6 %) and VIA (sensitivity: 74.2–79.4 %, specificity: 85.2–85.8 %), making it the preferred diagnostic option in many clinical settings [9].

Significant technological advancements have been made in capturing high-quality cervical images during visual inspection, yet specific challenges remain. Colposcopes, for instance, offer magnified visualization through specialized lenses [10], improving diagnostic accuracy; however, their high cost restricts their use in resource-limited settings. Cervicography, which captures images for remote evaluation by a professional, also presents limitations due to delayed diagnoses, which can hinder timely and effective treatment [11].

This article presents one of the key findings from the CITOBOT-COL translational research project, a large-scale initiative to enhance and validate CITOBOT as a portable device for cervical cancer screening. The project integrates an AI for classifying cervical lesions through advanced imaging. We detail the comprehensive technological development of the device, guided by a human-centered design approach and smart prototyping. To achieve this, we convened an interdisciplinary team that engaged in ideation and prototype development, utilizing the Design Thinking methodology to ensure the device met the specific needs of healthcare providers and patients.

## 2 Materials and methods

The development of the CITOBOT device followed a user-centered Design Thinking [12], [13] methodology combined with smart prototyping [14] techniques, prioritizing empathy, collaboration, and iterative feedback from end-users. The objective was to create a device that met clinical needs, was easy to use, and provided patient comfort, particularly in resource-limited settings.

### 2.1 Human-centered design and iterative process

Between 2022 and 2023, recurring focus groups guided the development of CITOBOT, essential in identifying user needs, challenges, and preferences regarding the device's functionality, usability, and design. By directly engaging with these diverse groups, we gained valuable insights into the functional and ergonomic requirements for an effective cervical cancer screening device. The focus groups consisted mainly of physicians experienced in cervical cancer prevention, gynecology specialists, nurses, and potential patients. Additionally, software and AI experts in computer engineering participated, as well as public health professionals familiar with the care cycle in primary health centers.

Key issues such as ease of use for medical staff, patient comfort, and integration with existing clinical workflows were highlighted. These discussions shaped the device's design, emphasizing the need for it to be easy to manipulate while maintaining patient comfort.

After identifying the challenges, the team developed multiple design prototypes addressing specific user issues. The iterative process, rooted in Design Thinking, allowed for rapid testing, refinement, and real-time feedback from stakeholders. This cycle ensured that each version of CITOBOT was aligned with real-world needs, optimizing the final device for user comfort and operational efficiency.

### 2.2 Smart prototyping

Smart prototyping was integral to the development process, enabling rapid fabrication and testing of CITOBOT prototypes. 3D printing technologies, including Selective Laser Sintering (SLS) and Fused Deposition Modeling (FDM), were used to produce detailed prototypes from biocompatible materials. This allowed the team to quickly assess design elements such as handle ergonomics, camera positioning, and opening mechanisms, making real-time adjustments as necessary. Computer-aided design (CAD) software, particularly SolidWorks, was used to refine prototypes precisely. The smart prototyping approach also integrated AI algorithms to enhance imaging capabilities, aiding in detecting cervical lesions.

### 2.3 Key specifications addressed

Several critical specifications were identified and iteratively refined throughout the development process:

− *Camera positioning and visualization:* A 5-megapixel USB camera optimized for precise cervical imaging was incorporated. Separating the LED lights from the camera minimized image distortion and enhanced visualization [15].
− *Ergonomics and usability:* The handle was designed with a 110-degree ergonomic angle to allow healthcare providers to manipulate it easily. The handle and ratchet system were tested for user comfort and practicality during procedures.
− *Biocompatible materials:* The reusable handle and single-use components, including the working channel and opening device, were fabricated from biocompatible resins and polymers, similar to surgical-grade silicone, ensuring patient safety and comfort.

### 2.4 Testing

Each design iteration underwent fabrication and testing in a simulated hospital laboratory, allowing the team to assess functionality and identify necessary adjustments to materials and components. Three potential designs were developed using 3D CAD software for testing with gynecological anatomical models. These designs were fabricated using a combination of 3D printing, CNC machining, and plastic injection molding technologies. Materials such as PLA, TPU, TPE, and biocompatible resins were selected for their durability and clinical suitability.

### 2.5 Design evaluation and integration

The images captured by the USB camera were displayed and stored via a custom-developed application, allowing for image capture alongside patient data. Key specifications, outlined in Table 1, guided the design revisions and decision-making process throughout the development of CITOBOT, ensuring that the device met clinical and practical needs in resource-limited settings. [20].

**Table 1:** Primary design specifications considered.

Design specification		Rationale
Visual area	2.5 – 3 cm	Corresponding to the average cervical diameter, ensuring adequate visualization during examination[16].
Average vaginal cavity distance	10.04 cm	Representing the average length of the vaginal cavity, with variation depending on individual anatomical characteristics[17].
Working distance from the camera to the cervix	3 – 5 cm	The optimal range for the precise focus of the USB camera on the cervix ensures clear imaging.
Camera diameter	0.8 mm	Representing the minimum diameter of the USB camera, designed to ensure compactness and ease of insertion
Compatibility with other devices	Android 5.0 + / Windows PC / MAC	Compatible with Android 5.0 + , Windows P.C., and MAC platforms, enabling seamless camera connectivity through a custom application interface.
Biocompatibility of materials	ISO 10993–5, ISO 10993–10	Compliant with ISO 10993–5 and ISO 10993–10 standards, ensuring biological safety through in vitro cytotoxicity testing[18] and skin sensitization testing[19].
Instrument Compatibility	Swabs, acetic acid application, forceps	It is designed for seamless integration with swabs, acetic acid applicators, and forceps, allowing for efficient manipulation of various instruments during the procedure.
Risk level	Class IIA – Moderate risk	Classified as Class IIA – Moderate risk, by INVIMA regulations, based on factors such as duration of body contact, degree of invasiveness, and local versus systemic effects[20]

The specific parameters considered in this process are detailed in Table 2 [20].

**Table 2:** Validation parameters for volunteer patients before testing.

Parameter	Details
Electrical safety	Powered by 5 VDC via USB, providing a stable energy supply to the camera and LED lighting system.
Software	Wi-Fi-enabled application supports secure login, volunteer data entry, real-time image visualization, and storage.
Usability	Optimized for intuitive operation and efficient management by healthcare providers, ensuring seamless integration into clinical workflows
Disinfection	Pre-procedural sterilization is required and designed for single patients only, ensuring disposal after each use.

### 2.6 Data-driven UX

The continuous improvement of CITOBOT v4 was guided by applying data-driven UX principles. i) Device components were optimized through A/B testing on the overall design, handle ergonomics, and the placement of the camera and LED lights. This approach identified which versions provided a more comfortable and efficient experience for both healthcare professionals and potential patients. ii) The software's user interface was personalized to adapt to physicians' usage patterns in cancer prevention services, and iii) the time required for each step in the screening process with CITOBOT was optimized to enhance efficiency for its future implementation in real-world settings.

### 2.7 Methodological framework for developing and validating AI and software systems

In addition to the smart prototyping of the CITOBOT device, we developed AI and software for cervical cancer risk classification. The AI design involved several key phases: exploration and experimentation with initial models, creation and preparation of an image bank with categorized and optimized data, model selection and refinement using advanced techniques such as Transfer Learning, validation with training and test data, implementation within a programming environment, and final evaluation using performance metrics to ensure its real-world applicability.

The software development process similarly followed essential phases: requirements analysis and planning to establish the software’s objectives and functionality, architecture and user interface design for optimal usability and scalability, coding and integration to build and connect modules, testing and debugging to detect and resolve errors, deployment in the target environment, and a final evaluation phase with user feedback to confirm functionality and reliability, making the software fully operational and user-centered.

## 3 Results

Through four design iterations, we developed and refined CITOBOT v4, a transvaginal and portable device. The prototypes were validated through focus groups and testing conducted by physicians experienced in cervical cancer prevention, gynecology specialists, nurses, potential patients, software and AI experts in computer engineering, and public health professionals. These sessions used various anatomical models in the Simulated Hospital Laboratory at Pontificia Universidad Javeriana Cali, Colombia. Feedback was used to assess and refine the device's functionality, primarily focusing on capturing high-quality cervix images. Currently, CITOBOT v4 has two patents pending: 1) Utility Model Patent in Colombia (NC2024/0008763, filed on June 30, 2024) and 2) PCT (International Application No. PCT/IB2024/057963, filed on August 16, 2024). Each prototype is described below:

### 3.1 CITOBOT v1

**Fig. 1:**
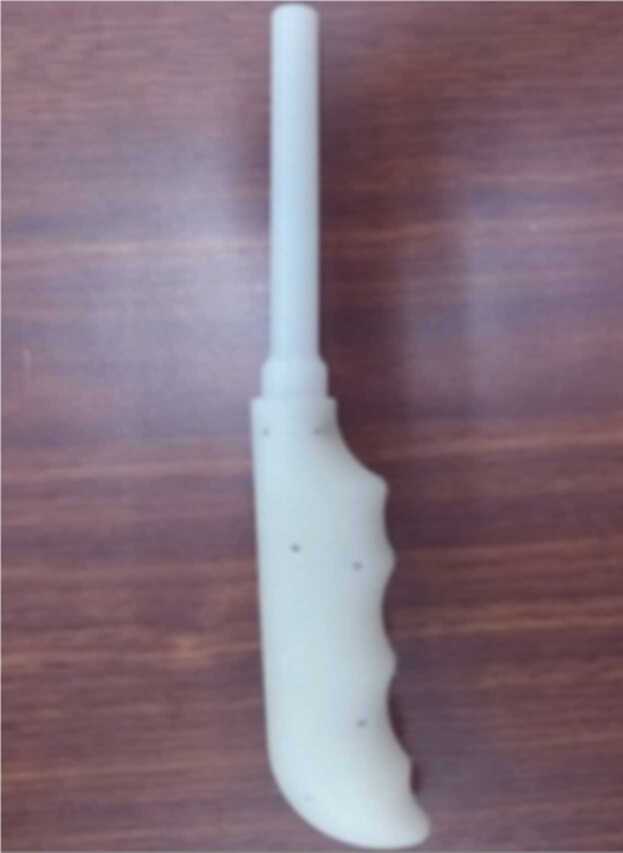
CITOBOT V1.

This prototype emerged in 2019 after the search for a more accessible solution to improve cervical cancer screening coverage within the Ladera Health Network (Red de Salud Ladera ESE) in Cali. This public health network serves vulnerable populations [21]. The device was conceived as a portable unit initially composed of two sections, which are detailed in Fig. 1:

1. USB Camera Protector: This slim, elongated component is encased in a disposable sheath designed to securely hold the USB camera at its tip, ensuring sterility during use.
2. Handle: This sturdier component functions as a grip and control interface, featuring buttons for capturing images and adjusting light intensity. The USB camera connects to an external display, such as a smartphone, tablet, or computer, to provide real-time visualization of the cervix. Both sections were fabricated from Teflon and machined using CNC technology.

This study explored the possibility of avoiding the use of a conventional speculum. However, the results from focus group discussions and tests conducted on anatomical models in a simulated hospital laboratory led us to conclude that it was necessary to integrate a mechanism to insert and hold the vaginal walls. This addition would provide the device with the required field of view for proper observation of the cervix [15].

### 3.2 CITOBOT v2

**Fig. 2:**
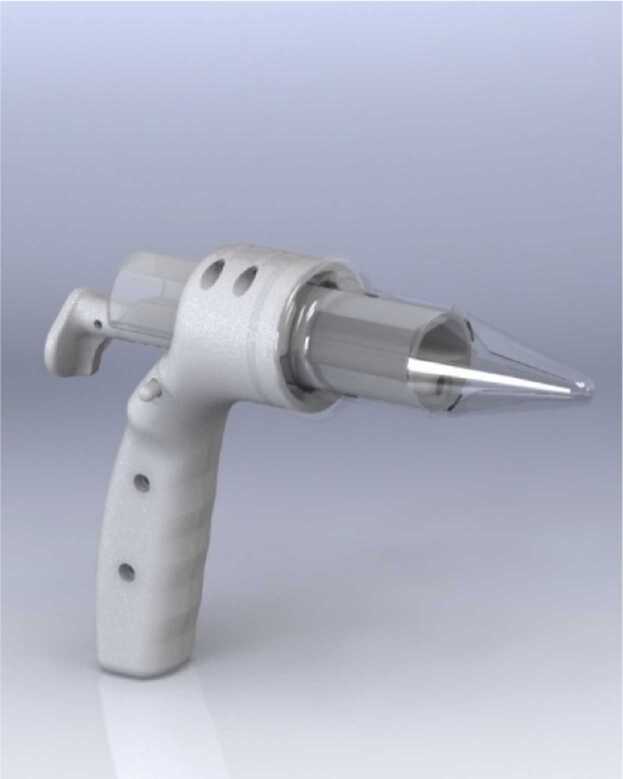
CITOBOT v2.

The v2 prototype (Fig. 2) was designed with three essential components:

1. Handle with 5-Megapixel USB Camera: The camera was integrated into the opening mechanism via a ratchet system and featured a lever for controlling the expansion of the flexible section, along with a button to lock and unlock the lever. The ergonomic handle was angled at approximately 110 degrees relative to the working channel and included assembly tabs for attaching the other components. The lever was operated with the thumb, and to retract it, the button was pressed while the thumb and index finger pulled the lever back. This handle component was reusable, whereas the other two were single-use per patient.
2. Instrument Working Channel: This single-use component allowed for the insertion of a brush for HPV sample collection, the application of contrast agents such as acetic acid or Lugol, and cleaning tools like gauze or swabs. It was attached to the handle's ratchet system and featured a protective lens to shield the camera from contamination.
3. Opening Device: The opening device was produced using an injection molding process that combined polymix TPE and LDPE. The latter material, like surgical-grade silicone used in menstrual cups, was selected for its comfort and ability to improve cervical visualization.

Regarding the materials used, the handle was manufactured from Teflon and machined using CNC technology, while the working channel was produced through 3D printing using biocompatible material. The opening device was created using the previously mentioned injection molding process. This design was inspired by the goal of enhancing comfort and exploring the potential for improved cervical visualization. However, during testing on various anatomical models, the device failed to meet the expectations of gynecologists and nurses due to difficulties in adequately opening the vaginal canal. The device could not withstand the pressure of the vaginal walls and did not provide sufficient ergonomics for the practitioners performing the procedure.

### 3.3 CITOBOT v3

**Fig. 3:**
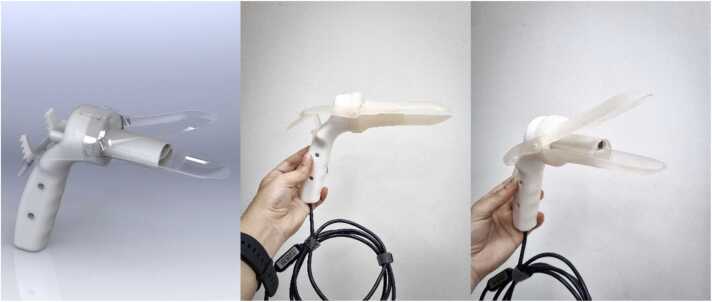
CITOBOT v3. Render in SolidWorks and fabricated prototype.

The three parts from the previous design were retained for this new prototype, but significant modifications were made to the opening mechanism and the materials used. In the handle, the design was updated by replacing the lever with a ratchet mechanism, which adjusts to the opening device like a pivot and is operated using the thumb. Additionally, the biocompatible resin was chosen instead of TPE, providing greater rigidity to hold the vaginal walls effectively. This prototype consists of the following parts, as illustrated in Fig. 3.

1. Handle with 5-Megapixel USB Camera: The handle is designed to encase the USB camera fully. In this iteration, the LED lights were deliberately separated from the camera to avoid interference with the visual angle and to minimize reflections or image distortion. The handle is positioned at an approximate 110-degree angle for ergonomic use and was fabricated from Teflon for durability. This part is reusable, while the other two components are designed for single use per patient.
2. Instrument Working Channel: The working channel retained the core design principles of the previous prototype, undergoing no significant changes and ensuring compatibility with existing tools and procedures.
3. Opening Device: In this version, the opening device was fabricated using 3D printing and biocompatible resin. This material, like the plastic used in conventional speculums, was selected for its durability and ability to improve cervical visualization during the procedure.

This version was designed to prioritize patient comfort. However, during the feedback process for the third version, in focus groups and tests on anatomical models, it became evident that the device did not provide adequate cervical opening with the push mechanism. Additionally, the working channel was too narrow, complicating the manipulation of swabs and forceps, the application of acetic acid, proper cleaning, and the collection of HPV DNA samples.

### 3.4 CITOBOT v4

**Fig. 4:**
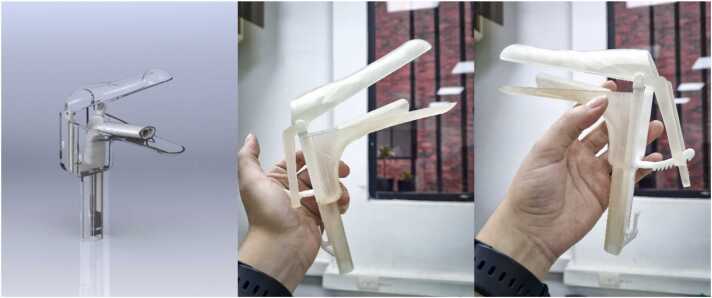
CITOBOT v4. Render in SolidWorks and fabricated prototype.

The v4 prototype featured a simplified configuration that included only the handle, integrating the camera mount and the opening device, as shown in Fig. 4.

1. Handle with Opening Mechanism: This component serves as the handle, allowing the device to be securely held and maneuvered during the procedure while also facilitating the opening of the vaginal walls. It comprises two parts: the lower and upper sections, assembled using two ratchets. The lower section houses the camera channel, angled at approximately 20 degrees to enhance cervical visualization. The camera fits snugly within this channel, and the ratchets ensure enough space to use forceps and other essential tools during the procedure. These parts were 3D printed using biocompatible resin, providing the necessary rigidity and a texture similar to a conventional speculum.
2. Ratchets: These mechanisms allow for the assembly of the two sections and enable the gradual, controlled opening of the device. The device can be adjusted using the thumb, mimicking the operation of a traditional speculum. The ratchets can also be adjusted vertically using the thumb to regulate the opening during the procedure.
3. Megapixel USB Camera: The camera is positioned within a second handle and accompanied by LED lights for optimal visualization. It remains fixed during the procedure but can be attached or detached as necessary. Unlike the other components, the camera is reusable. The ratchets and this component were fabricated from polyamide 12 using SLS (Selective Laser Sintering) 3D printing technology.

In this final version, the core design concept from the previous iteration was retained, but the material and opening mechanism were modified. Ratchets were incorporated to allow for one-handed manipulation, and the camera was positioned slightly higher to improve visualization of the cervix.

With CITOBOT v4 validated by users, the authors conducted a clinical case series study, evaluating its acceptability with 20 volunteer patients at ‘Unicáncer,' Cali, Colombia. The results showed high overall acceptability of the device, with measurements assessing comfort, safety, pain, and material quality (results currently under peer review). Unlike the traditional speculum, users appreciated that CITOBOT requires rotational movements inside the vaginal cavity to obtain a complete view of the cervix, which was positively received.

Finally, CITOBOT v4 was designed to integrate two additional technological developments: AI and specialized software.

### 3.5 Design, development, and validation of AI for cervical cancer risk classification

The AI integration in CITOBOT v4 underwent several critical phases. Initially, a database of 1310 cervical images was compiled from sources such as the National Cancer Institute (USA) and WHO. Various machine and deep learning models and techniques were tested, focusing on image segmentation to reduce noise and enhance performance in relevant areas. A specific image bank of 1621 images, provided by a hospital in Cali, Colombia, was then developed, with images classified by experts into "negative for lesion" and "at risk" categories. Of these images, 80 % were used for training the model and 20 % for validation. Preprocessing techniques such as segmentation (using the SAM model) and data augmentation—including rotations and lighting adjustments—were applied to improve the model's generalization capacity in real clinical environments.

During the design phase of the AI model, multiple architectures were tested, with the Inception V3 network ultimately chosen for its ability to capture information at diverse scales and its high performance in complex image classification. This model was optimized using Transfer Learning and Fine Tuning, achieving approximately 90 % sensitivity and specificity in cervical cancer risk classification. Model validation involved data partitioning (80 % for training and 20 % for validation) and cross-validation, using metrics like accuracy, sensitivity, specificity, and AUC to ensure robust performance. (Results currently under peer review).

### 3.6 Software integration

The AI is embedded within the "CITOBOT – mobile and web application" software (registered in Colombia #13–98-111 on 04/03/2024). This software guides healthcare providers through cervical cancer screening, transmitting cervical images to the AI system, which responds within seconds. Notably, the software can operate offline, allowing seamless use in areas with limited connectivity. It also includes features for tracking patient screening history and retraining the AI model as necessary.

Python with TensorFlow and Keras was used for implementation, employing optimization techniques such as EarlyStopping and dynamic learning rate adjustment. CITOBOT was deployed on mobile devices using TensorFlow Lite, enabling fast, offline predictions for practical use in diverse healthcare settings.

## 4 Discussion

This study represents a crucial example of technological innovation in Colombia, where investment in science and healthcare technology remains limited compared to higher-income countries. The development of the CITOBOT v4 device in a resource-constrained environment highlights the importance of interdisciplinary collaboration and a user-centered design approach to meet the unique needs of patients and healthcare providers.

The prototype of CITOBOT was described in 2020 [21]. To date, it is a device at Technology Readiness Level (TRL) 6, tested with volunteer patients for design acceptability, comfort, and safety. Additionally, CITOBOT v4 incorporates software and an AI system for cervical cancer risk detection, making it especially useful in resource-limited environments due to its portability and as a screening alternative that reduces the time required to begin diagnosis and treatment.

In line with previous studies, our research also emphasizes the importance of technological devices in healthcare and cervical cancer detection, particularly in healthcare facilities that face challenges accessing highly trained personnel, colposcopes, cervicography, or HPV DNA testing [22], [23], [24]. Imaging for cervical cancer detection provides a highly accessible alternative, mainly when computers or mobile phones can visualize the cervix and analyze the images with artificial intelligence [25], [26].

In this article, we highlight the smart prototyping based on a user-centered approach, which led to a new device that enables the capture of cervical images while facilitating direct observation, as well as the manipulation of cleaning instruments, acetic acid application, and sample collection. The user-centered design ensured that patient acceptability was prioritized. Furthermore, the decision to incorporate affordable materials and integrate mobile technology reflects the commitment to creating a solution that can be widely adopted in regions where access to high-cost medical equipment, such as colposcopes, is often limited.

Smart prototyping played a crucial role in the design of CITOBOT v4. This approach integrates advanced digital technologies, such as 3D printing, to quickly iterate designs based on real-time feedback from users and stakeholders. By employing smart prototyping, our team created a functional device that meets the specific needs of users. 3D printing allowed for the rapid fabrication of prototypes, facilitating quick adjustments to design flaws and material choices. This method accelerated the development process and ensured the final product was highly adaptable and suitable for use in regions with limited access to traditional and expensive medical equipment. Smart prototyping proved essential in bridging the gap between technological innovation and practical application.

Regulatory compliance was also a key consideration in the development process. CITOBOT v4 meets biocompatibility standards (ISO 10993–5 and ISO 10993–10), ensuring the device's biological safety. In Colombia, CITOBOT v4 is classified as a Class IIA device under the National Institute for Food and Drug Surveillance (INVIMA by its initials in Spanish) regulations, meeting moderate-risk standards that focus on safety and efficacy [27]. CITOBOT faces the challenge of aligning with international regulatory frameworks to facilitate its adoption in other markets, including the U.S. Food and Drug Administration (FDA) and the European Medical Device Regulation (MDR).

In the United States, the FDA classifies medical devices into three classes (I, II, and III) based on risk. Class II devices, like CITOBOT, generally require a premarket notification [510(k)] to demonstrate substantial equivalence to a legally marketed device regarding safety and efficacy [28]. Additionally, the European Medical Device Regulation (EU MDR 2017/745) classifies devices based on risk, with Class IIa devices requiring the involvement of a notified body to assess conformity before commercialization. This process includes a rigorous evaluation of technical documentation and clinical testing to ensure safety and performance [29]. Aligning CITOBOT v4 with these international regulatory frameworks would facilitate its adoption in diverse markets and ensure it meets global standards for safety and efficacy in smart medical devices.

The authors faced various challenges in designing CITOBOT v4, not only because public investment in research and innovation is scarce but also due to the underdevelopment of Colombia's national medical technology industry. During the design process, we encountered issues related to the purchase and importation of supplies and the need for advanced technology for manufacturing. Despite these obstacles, the CITOBOT-COL project models how local efforts can generate solutions tailored to the specific needs of underserved populations. The device holds significant promise for improving cervical cancer detection, with the potential to be a low-cost, portable alternative to traditional methods.

The authors recognize that the value of this article lies in its significant contribution to technological development, which is focused on creating a new device for cervical cancer screening that has led to pending patent applications. This work highlighted practical aspects of the design, ergonomics, and customization of CITOBOT v4, directly addressing the need for accessible solutions in resource-limited settings. The innovation resided in an iterative technological process guided by user-centered design principles and data-driven UX, which optimized the device's functionality and usability in real-world situations. Our primary objective was not to generate new knowledge but to emphasize the device's potential impact on medical practice. In a field where the technological development of many patented devices is not openly known, this work stands out as a detailed and highly relevant applied technological contribution to advancing health technology rather than as a theoretical addition to fundamental research.

Finally, the design of CITOBOT v4 highlights the critical importance of driving innovation in countries with limited resources, offering an effective solution to enhance cervical cancer screening and potentially save lives in vulnerable communities. Future steps include rigorous clinical validation studies, such as assessments of sensitivity and specificity, reproducibility, concordance, and validation across diverse populations. Meanwhile, the findings presented in this article serve as a pivotal example of technological advancement in Colombia, where investment in health science and technology remains relatively limited. The development of CITOBOT v4 in this context underscores the value of interdisciplinary collaboration and a user-centered design approach to meet the specific needs of patients and healthcare providers.

## CRediT authorship contribution statement

**Paula C. Bermúdez:** Writing – review & editing, Writing – original draft, Visualization, Validation, Supervision, Methodology, Investigation, Funding acquisition, Formal analysis, Data curation, Conceptualization. **Marcela Arrivillaga:** Writing – review & editing, Writing – original draft, Visualization, Validation, Supervision, Project administration, Methodology, Investigation, Funding acquisition, Formal analysis, Data curation, Conceptualization. **Bleider Arizala:** Writing – review & editing, Validation, Investigation. **Daniela Morales:** Writing – review & editing, Validation, Investigation. **Mérida Rodríguez-López:** Writing – review & editing, Supervision, Methodology, Investigation, Conceptualization. **Maria del Mar Torres:** Writing – review & editing, Validation, Supervision, Investigation. **Daniela Neira:** Writing – review & editing, Validation, Investigation. **Hernán Darío Vargas-Cardona:** Writing – review & editing, Supervision, Methodology, Investigation, Data curation. **Juan Pablo García-Cifuentes:** Writing – review & editing, Software, Methodology, Conceptualization.

## Declaration of Generative AI and AI-Assisted Technologies in the Writing Process

During the preparation of this manuscript, the author(s) used ChatGPT-4.0 to assist with language clarity and stylistic editing. The author(s) critically reviewed, edited, and approved all AI-generated content to ensure scientific accuracy, taking full responsibility for the final manuscript content.

## Declaration of Competing Interest

We declare that we have no known competing financial interests or personal relationships that could have appeared to influence the work reported in this paper.
